# Functionality in Electrospun Nanofibrous Membranes Based on Fiber's Size, Surface Area, and Molecular Orientation

**DOI:** 10.3390/membranes1030249

**Published:** 2011-08-26

**Authors:** Hidetoshi Matsumoto, Akihiko Tanioka

**Affiliations:** Department of Organic and Polymeric Materials, Tokyo Institute of Technology, 2-12-1 Ookayama, Meguro-ku, Tokyo 152-8552, Japan

**Keywords:** nanofiber, membrane, electrospinning, nanosize effect, high surface area, molecular orientation

## Abstract

Electrospinning is a versatile method for forming continuous thin fibers based on an electrohydrodynamic process. This method has the following advantages: (i) the ability to produce thin fibers with diameters in the micrometer and nanometer ranges; (ii) one-step forming of the two- or three-dimensional nanofiber network assemblies (nanofibrous membranes); and (iii) applicability for a broad spectrum of molecules, such as synthetic and biological polymers and polymerless sol-gel systems. Electrospun nanofibrous membranes have received significant attention in terms of their practical applications. The major advantages of nanofibers or nanofibrous membranes are the functionalities based on their nanoscaled-size, highly specific surface area, and highly molecular orientation. These functionalities of the nanofibrous membranes can be controlled by their fiber diameter, surface chemistry and topology, and internal structure of the nanofibers. This report focuses on our studies and describes fundamental aspects and applications of electrospun nanofibrous membranes.

## 1. Introduction

**Figure 1:**
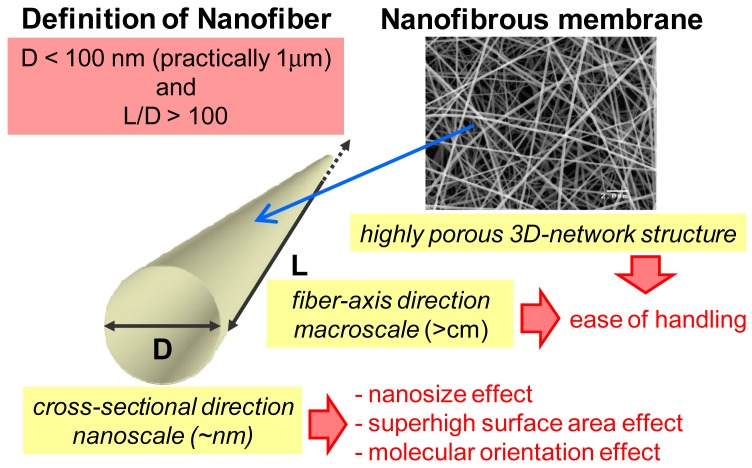
Characteristics of nanofiber and nanofibrous membrane.

Ultrafine fibers, called “nanofibers” are a unique nanomaterial because of the nanoscaled dimensions in the cross-sectional direction and the macroscopic length of the fiber axis (see Figure 1). Therefore, nanofibers have both the advantages of functionality due to their nanoscaled structure and the ease of manipulation due to their macroscopic length. In addition, three-dimensional nanofiber network assemblies (nanofibrous membranes or fabrics) provide good mechanical properties and good handling characteristics.

Electrospinning is a versatile method based on an electrohydrodynamic process for forming continuous thin fibers ranging from several nanometers to tens of micrometers and can be used for the one-step forming of thin fibrous membranes [1,2,3,4,5]. The variety of materials, such as the polymer-solvent systems and polymerless sol-gel systems can be electrospun [6]. Electrospun nanofibrous membranes with high surface areas have drawn significant attention for their practical applications, such as high-performance filter media, protective clothes, composites, drug delivery systems, scaffolds for tissue engineering, sensors, and electronic devices [2,3,4,5]. The functionalities of the nanofibers or nanofibrous membranes are based on their nanoscaled-size, high specific surface area, and high molecular orientation, and they can be controlled by their fiber diameter, surface chemistry and topology, and internal structure of the nanofibers. In addition, processing innovations to improve not only the controlling of morphologies but also the production capacity of electrospun nanofibers and nanofibrous membranes are in progress. In particular, the high-throughput electrospinning systems are an ongoing development (e.g., multi-needle and needleless processes) [7].

This review describes our fundamental studies on controlling of fiber diameter, surface chemistry and topology, and internal structure of the nanofibers by electrospinning and highlights our efforts toward the applications of nanofibrous membranes such as ion-exchangers, air filters, and antimicrobial materials.

## 2. Control of Structure of Nanofibers and Nanofibrous Membranes

### 2.1. Control of Size, Internal Structure, and Surface of Nanofiber

Many material parameters and process parameters have important effects on both the electrospinning process and the resulting fiber morphology [8]. The fiber diameter depends on the solution properties (e.g., viscosity, conductivity, surface tension, permittivity, and boiling point) and/or operating conditions (e.g., applied voltage, spinneret-to-collector distance, and flow rate) summarized in Table 1.

**Table 1:** Control parameters for morphology and diameter of electrospun fibers (Adapted from [5]).

**Solution properties**

Viscosity (molecular weight of polymer and concentration)
Electric conductivity
Solvent properties (surface tension, boiling point, polarity, and permittivity)

**Operating conditions**

Applied voltage (typically from several kV to several 10 kV)
Distance between spinneret and collector (typically from several cm to 50 cm)
Feeding rate of polymer solution
Spinneret (inner diameter, shape, and material)

**Surrounding conditions**

Temperature
Humidity

**Figure 2:**
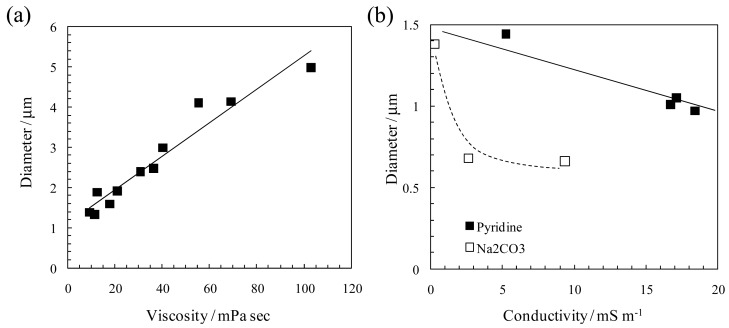
(**a**) Effect of solution viscosity on the diameters of the electrospun phenolic-resin fibers. Solution viscosity was changed by polymer concentration. (**b**) Effect of solution conductivity on diameters of the electrospun phenolic-resin fibers. Additives to the spinning solution are pyridine and sodium carbonate (Adapted from [9]).

Practically, the viscosity and electric conductivity of the spinning solutions are crucial factors for controlling the fiber diameter. Figure 2 shows the typical effects of the solution viscosity and conductivity on the diameter of the electrospun fibers [9]. In Figure 2a, the solution viscosity was changed by polymer concentration and the more viscous solution tends to form a thicker fiber. The increase in the entanglement of polymer chains caused by increasing the polymer concentration and/or molecular-weight of the polymer contributes to the formation of the fibrous structure. In addition, higher-molecular-weight polymers improve the electrospinnability from the lower viscosity solution and are effectively fabricated into thinner and homogenous fibers by electrospinning [10]. The fiber diameter also decreases with an increase in the solution conductivity due to the addition of a small number of electrolytes (Figure 2b, pyridine and sodium carbonate were used as the organic and inorganic electrolytes, respectively). The adequate conductivity solution enhances electrostatic repulsion force on the surface of the jet during electrospinning, and consequently, the fiber diameter decreases [9]. However, the high-conductivity solution prevents electric-field induced charging of the solution, and consequently show a low electrospinnability.

**Figure 3:**
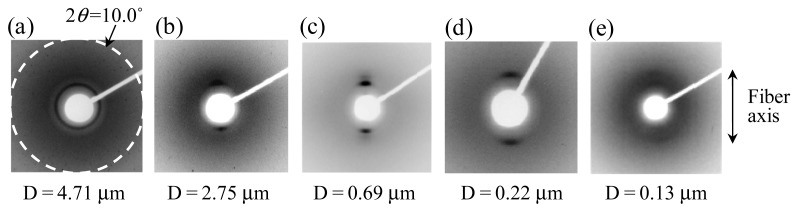
Effect of fiber diameter on structure formation and orientation of liquid crystalline polymer in electrospun nanofibers; the WAXD patterns of the uniaxially aligned electrospun LCP fibers with diameters (D) of (**a**) 4.71 μm, (**b**) 2.75 μm, (**c**) 0.69 μm, (**d**) 0.22 μm, and (**e**) 0.13 μm (Adapted from [14]).

Many properties of the nanofibers, such as the mechanical, electrical, and optical properties depend not only on the intrinsic properties of a polymer, but also on the internal structure of the fibers. Therefore, control of the internal structure and molecular orientation of the nanofibers has been required for improvement of their properties. Recently, some researchers reported that electrospinning is a useful process for controlling the crystal morphology and molecular orientation of poly(ethylene oxide) (PEO) and polyoxymethylene (POM) nanofibers [11,12]. Our previous study also showed that the electrospun poly(vinylidene fluoride) (PVDF) nanofiber has an oriented crystalline region (the polar *β*-phase crystal is dominant) along the fiber axis [13]. In addition, we reported that the formation and orientation of the ordered structure in the electrospun liquid crystalline polymer (LCP) fibers could be controlled by the fiber diameter during electrospinning [14]. Figure 3 shows the wide-angle X-ray diffraction (WAXD) patterns of the uniaxially aligned electrospun LCP (main-chain LC polyester, BB-5(3-Me)) fibers with various diameters. The patterns include two sharp reflections with smectic layer spacings of 24.0 and 17.0 Å. The reflection patterns are ring-shaped for the thickest fiber with the diameter (D) = 4.71 μm (Figure 3a), and the sharp reflections are concentrated on the meridian for the thinner fibers (Figure 3b–d). The concentrated reflections indicate that the layer normal of the smectic layer is arranged parallel to the fiber axis in the electrospun LCP fibers. The clearest reflections were found for the fiber with D = 0.69 μm (Figure 3c). On the other hand, such clear reflections have not been observed for the thinnest fiber with D = 0.13 μm (Figure 3e). Possibly, fast solidification of a thinner jet during electrospinning due to the high specific surface area prevents formation of an ordered internal structure. In addition, the incorporation of nanostructures, such as carbon nanotubes (CNTs) and functional nanoparticles (e.g., TiO_2_ or magnetic nanoparticles), within the electrospun nanofibers develop their internal structures and significantly improve their properties [15].

**Figure 4:**
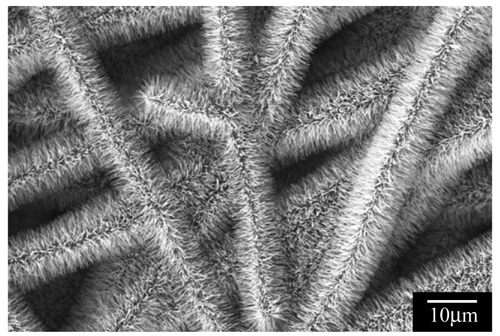
Scanning electron micrograph of zinc oxide nanowires densely grown on the surface of carbon nanofibrous membranes (Adapted from [17]).

The nanofiber surface is a suitable platform for the functionalization and hybridization by chemical compounds and nanostructures, respectively. The superhigh surface area of nanofibers allows the maximization of the functions from the surface functional groups (e.g., ion-exchange groups) and nanostructures (e.g., CNTs [16], ZnO nanowires (Figure 4) [17], and other 1D nanostructures and nanopaticles). This surface functionalization of the nanofibers will lead to a significant expansion of their applications. One of the surface fictionalization examples of the electrospun nanofibers will be described in Section 3, *i.e.*, ion-exchangers.

### 2.2. Pore structure of Nanofibrous Membranes

**Figure 5:**
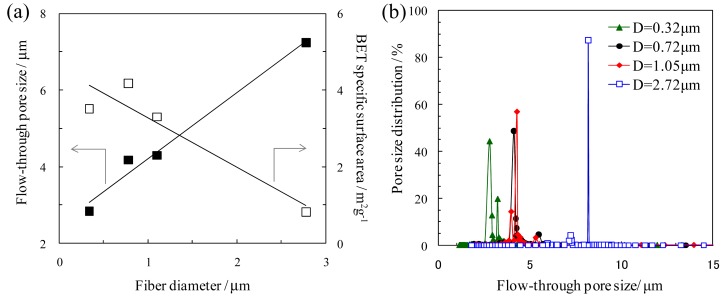
(**a**) Effect of fiber diameter on flow-through pore size between fibers and specific surface area of electrospun silica nanofibrous membranes; (**b**) Pore size distribution of electrospun silica nanofibrous membranes with various fiber diameters (D). The average pore size and pore size distribution were characterized by the bubble-point method (ASTM F316-86, JIS K3832) (Adapted from [18]).

Electrospinning produces highly porous nanofiber network structures with interconnected flow-through pores. The typical trend for the effect of the fiber diameter on the porous structure is shown in Figure 5 [18]. The average pore size between nanofibers (*i.e.*, flow-through pore size) decreased with a decrease in the fiber diameter, and the Brunauer–Emmett–Teller (BET) surface area of the nanofibrous membranes increased with a decrease in the fiber diameter (Figure 5a). The size distribution of the flow-through pores was very narrow (Figure 5b). This is one of the remarkable features of electrospun nanofibrous membranes. In addition, the surface areas of the nanofibers can be further increased by the formation of intra-fiber pores. For example, it is possible to form intra-fiber pores (i) by phase separation based on evaporation of the solvent or in the presence of a vapor during electrospinning; and (ii) by electrospinning of the polymer blends or composites followed by selective removal of one component [3]. The intra-fiber pores or rough surfaces of the nanofibers, which increase the surface area and alter the kinetics of the adsorption/desorption or reaction, are advantageous for more advanced applications of the nanofibrous membranes (e.g., high-capacity adsorption membranes, highly-effective catalyst system, and rapidly chargeable/dischargeable electrodes).

## 3. Ion-Exchangers

### 3.1. Ion-Exchange Nanofibrous Membranes by Electrospinning

**Figure 6:**
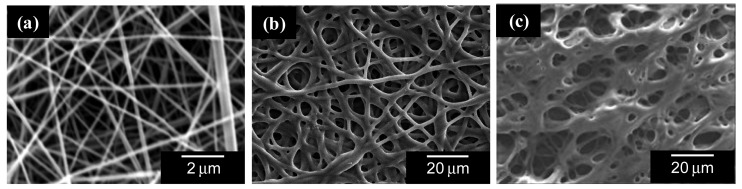
Surface scanning electron micrographs of the typical ion-exchange NFMs: (**a**) chitosan NFM (Adapted from [23]); (**b**) sulfonated PS NFM (Adapted from [21]); and (**c**) quaternized P4VP NFM (Adapted from [21]).

Recently, the scope of ion-exchange technology has widened into the fields of biotechnology, pharmaceutical processing, producing ultrapure water for the semiconductor industry, catalytic conversion processing, and battery technologies [19]. In particular, various ion-exchange fibers have been successfully used due to their high specific surface areas, good mechanical properties, good handling characteristics, and flexibility for processing into diverse forms. The introduction of ionic functional groups into the nanofibers is a promising option to provide novel ion exchangers with a high exchange capacity. In general, polymer solutions with a high electric conductivity (e.g., polyelectrolyte solution) show a low electrospinnability, because the solution conductivity prevents electric-field induced charging of the solution. Therefore, most of the approaches for forming ion-exchange nanofibers has relied on (i) the addition of water soluble and electrospinnable polymers to the spinning solution as the carrier, and (ii) electrospinning of a nonionic polymer or inorganic materials (e.g., sol-gel and carbon precursors) and successive chemical modification. We have reported the preparation of ion-exchange nanofibrous membranes (NFMs) by the electrospinning of biological polymers (chitosan [20]); electrospinning and successive chemical modification of synthetic polymers (polystyrene (PS) and poly(4-vinylpyridine) (P4VP) [21]); and electrospinning, successive carbonization and chemical modification (phenolic resin-based carbon [22]). The ionic biopolymer NFMs were prepared from polysaccharide/poly(ethylene oxide) (PEO) blended solutions. The ionic synthetic polymer NFMs, on the other hand, were obtained by the sulfonation of the as-spun PS NFMs and the quaternization of the as-spun P4VP NFMs. The physicochemical properties of the typical ion-exchange NFMs are summarized in Table 2. Figure 6 shows the surface scanning electron micrographs of the typical ion-exchange NFMs. These results show that the NFMs have a submicro-microscaled interconnected flow-through pore structure composed of nano-microscaled fibers, high porosity (>75%), and ion-exchange groups on and in the fiber (ion-exhange capacity = 0.8−5.4 mmol/g), which favorably compares to the reported capacity of 1–4 mmol/g for conventional ion-exchange materials [21]. The high BET specific surface area for the quaternized P4VP NFM (600 m^2^/g) is due to formation of the intra-fiber pores with a diameter of several nanometers during chemical modification of the P4VP NFMs [21].

**Table 2:** Physicochemical properties of ion-exchange nanofibrous membranes (NFMs) (Adapted from [21,23]).

**Material**	**Chitosan**	**Sulfonated PS**	**Quaternized P4VP**
Type (ion-exchange group)	anion-exchange (amino group)	cation-exchange (sulfonic acid group)	anion-exchange (tertiary pyridyl and quaternary pyridinium groups)
Ion-exchange capacity *1 [mmol/g-dry membrane]	5.4	1.3	0.8
Average pore size *2 [μm]	0.5	1.9	3.5
Membrane porosity *2 [%]	92	75	80
Through-pore specific surface area *2 [m^2^/g]	26	13	14
BET specific surface area *3 [m^2^/g]		2	600
Thickness [μm]	59	52	40

*1 Determined by potentiometric titration;

*2 Estimated by bubble-point method;

*3 Estimated by N_2_ adsorption/desorption experiments

**Figure 7:**
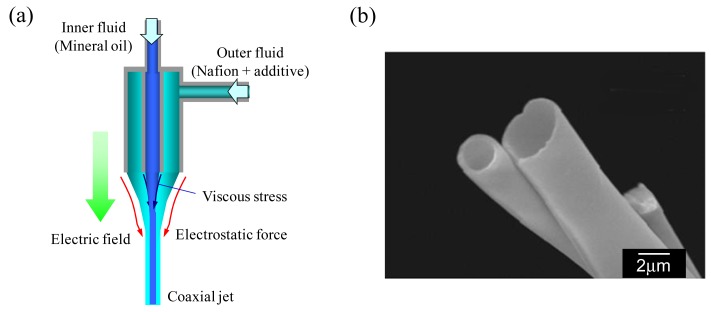
(**a**) Schematic diagram of co-axial nozzle for electrospinning; and (**b**) surface scanning electron micrographs of Nafion hollow microfiber prepared by co-axial electrospinning (Adapted from [24]).

More recently, we prepared microscaled Nafion (commercial perfluorosulfonate ionomer) hollow fibers by a two-fluid electrospinning process (Figure 7) [24]. The polyelectrolyte (ion-exchange) hollow fiber can be applied to micro-tubular fuel cells, micro-fluidic devices [25], and high-capacity membrane modules for water treatment.

**Figure 8:**
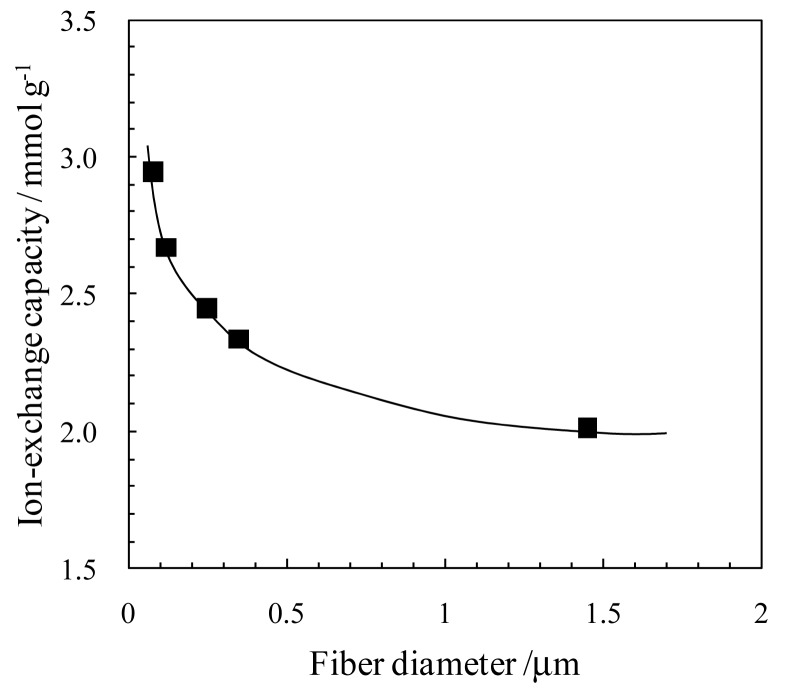
Effect of fiber diameter on ion-exchange capacity of surface sulfonated carbon NFMs (Adapted from [22]).

The typical trend for the effect of the fiber diameter on the ion-exchange capacity for surface-modified NFMs is shown in Figure 8. The capacity increased with a decrease in the fiber diameter. This is due to the surface area effect of the nanofibers. Elabad *et al.* also reported another nanosize effect on the ion conductivity of the ion-exchange nanofibers; the proton conductivity of the Nafion nanofibers sharply increased by decreasing the fiber diameter down to a nanometer–scale (the maximum conductivity of 1.5 S/cm for the nanofiber with a diameter of 400 nm is higher than that of 0.1 S/cm for the bulk film) [26]. This is due to the orientation of the ionic domains along the Nafion nanofiber axis. These results indicate that not only the surface functionalization, but also controlling of the internal structure can improve the properties of the ion-exchangers.

### 3.2. Catalytic Effect of Ion-Exchange Nanofiber on Water Splitting in Bipolar Membrane Electrodialysis System

**Figure 9:**
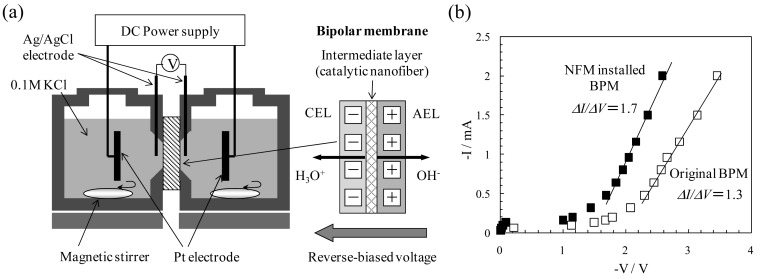
(**a**) Schematic diagram of apparatus for bipolar membrane (BPM) electrodialysis system under the reverse-bias condition. CEL: cation-exchange layer; AEL: anion-exchange layer. (**b**) Current–voltage characteristics of the BPMs, which consist of CEL/catalytic NFMs/AEL (Adapted from [28]).

Bipolar membranes (BPMs), which are composed of cation- and anion-exchange layers (CEL and AEL) joined together in series, show a water-splitting behavior under a reverse bias condition (Figure 9a). The BPM-based electrodialysis is an efficient process for generating acids and bases without by-products and is used for the recovery of acids and bases from wastewater, the purification of amino acids, and the recycling system for liquid-crystal display (LCD) manufacturing. The water splitting phenomenon occurs at the interface between the CEL and AEL. Therefore, the water-splitting capability of BPMs depends on their interfacial state [27]. In our previous study, the anion-exchange NFMs, *i.e.*, quaternized P4VP NFMs (see Table 2 and Figure 6c), were used as the intermediate layer of the BPM [28]. Figure 9b shows the current–voltage characteristics of the BPMs with the NFM installed (closed squares) and without any (open squares) in the intermediate region under reverse bias conditions. The electric-field-enhanced dissociation can be observed for an applied voltage higher than about 1 V, and the water dissociation is enhanced by the NFM layer. The Δ*I*/Δ*V* value increased from 1.3 to 1.7. The pH change in the compartments adjacent to the NFM layer-installed sample was greater than that of the uninstalled one. This is in good agreement with the current–voltage characteristics and suggests that the installed anion-exchange NFM layer enhanced the water dissociation in the intermediate region of the BPM. This result can be explained by the synergetic effect of the protonation–deprotonation reaction due to the anion-exchange group and the high BET specific surface area of the NFMs (600 m^2^/g, see Table 2). These results indicated that ion-exchange NFMs with a catalytic activity and a high surface area can improve the performance of the bipolar membrane-based electrodialysis processes.

## 4. Air Filters

**Figure 10:**
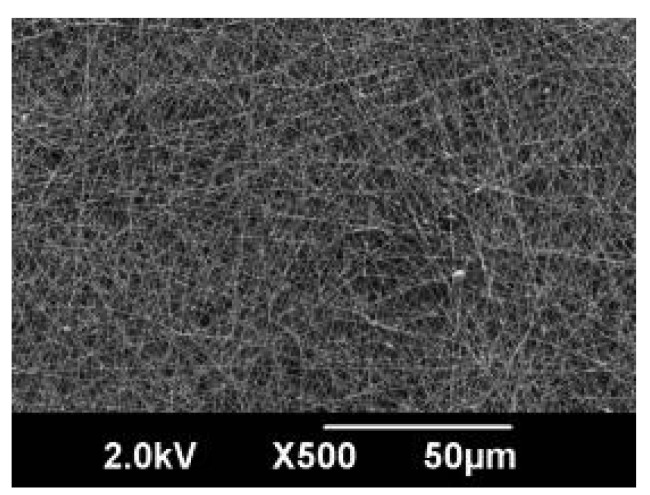
Surface scanning electron micrographs of typical electrospun PAN NFMs coated on glass fibrous nonwoven substrate. Average fiber diameter (D) = 90.4 nm, basis weight = 0.3 g/m^2^.

**Figure 11:**
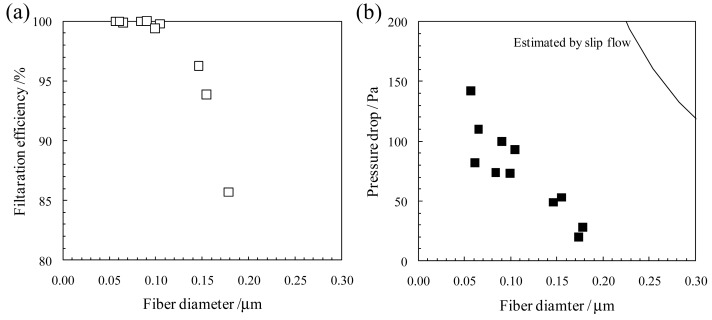
Effect of fiber diameter on (**a**) filtration efficiency and (**b**) pressure drop of the electrospun PAN NFMs. Basis weights of all the samples were fixed at 0.3 g/m^2^. All measurements were performed using particles with a diameter of 0.3 μm under a face velocity of 5.3 cm/s. The filtration efficiency and pressure drop of the glass fibrous nonwoven substrate are 11% and 5 Pa, respectively. Therefore, the contribution of the substrate to the filter performance is negligible.

Electrospun nanofibrous membranes have been very successfully applied in the field of air filtration because of their unique nanosize effect [4] (*i.e.*, aerodynamic slip at the nanofiber surface, called “slip flow” [29]). In general, fibrous filters are characterized by their collection efficiency and pressure drop. We have verified the effect of slip flow on the filtration efficiency and pressure drop for poly(acrylonitrile) (PAN) NFMs with thin fiber diameters (D = 50–180 nm) coated on a glass fibrous nonwoven substrate, as shown in Figure 10 [30]. All measurements were carried out according to JIS B9927. Figure 11 shows the filtration efficiency and pressure drop of the electrospun PAN NFMs as a function of the fiber diameter. The filtration efficiency, *η* = 1 − C_2_/C_1_ (where C_1_ and C_2_ are the number concentration of particles at filter upstream and downstream, respectively), increased with a decrease in the fiber diameter, and showed a value of *η* greater than 99% for fiber diameters of less than 100 nm. The pressure drop across the filter, ΔP, increased with a decrease in the fiber diameter. This is a general trend according to filtration theories [31]. To examine the effect of slip flow in detail, a theoretical prediction of slip flow was performed using Pich's approach [32]. The solid line in Figure 11b represents the calculated results based on Pich's equations for the pressure drop in the slip flow region (for a fiber of around 523 nm). The experimental results showed a similar trend for the calculated results, and the experimental ΔP values were much lower than the calculated ones considering the effect of aerodynamic slip at the nanofiber surface. These results support the fact that the slip flow mechanism is substantial for the NFMs with thin fiber diameters, and NFMs provide a promising platform for high-performance filter media with a higher filter efficiency and lower pressure drop.

Further improvement of the filtration performance will be possible. For example, the electret fibers due to their polar nature can enhance the collection efficiency for both charged and uncharged aerosols. The PVDF NFMs with a high *β*-phase content and intra-fiber pores [33] are expected to be a promising material as highly efficient filter media.

## 5. Antimicrobial Materials

Many antimicrobial agents, such as metallic and inorganic agents (e.g., silver, copper, zinc, and titanium dioxide), organic agents (e.g., quaternary ammonium and other cationic compounds), and natural and biological agents (e.g., hinokitiol, chitosan, antibiotics), have been applied to textile and membrane materials by various chemical and physical techniques for curing and preventing diseases for public health hygiene and antifouling in the biomedical industry [34]. The antimicrobial effect of the chitosan NFM mentioned above (see Table 2 and Figure 6a) is shown in Table 3 [35]. Two kinds of commercial fibrous membranes composed of microscaled fibers were tested for comparison. Unlike these controls, no bacteria could be detected on the chitosan NFM after incubation. In addition, the chitosan fibrous membrane with a thinner diameter is more effective. This is attributed to the high surface area of the NFM. This demonstrates that electrospun NFMs are a promising platform for enhancing the function of antimicrobial agents.

**Table 3:** Antimicrobial effect of fibrous membranes (Adapted from [35]).

	Bacterial count (cells/vial) *1			

	**Before incubation**	**After incubation**
Chitosan nanofibrous membrane	5.4 × 10^5^	0
Chitosan microfibrous membrane *2	5.4 × 10^5^	4.2 × 10^3^
Polyethylene terephtalate (PET) microfiber membrane *2	5.4 × 10^5^	2.4 × 10^5^

*1 Determined by Japanese Industrial Standard (JIS L1902) using *Staphylococcus aureus* (NBRC1237);

*2 Commercial fibrous membranes

**Figure 12:**
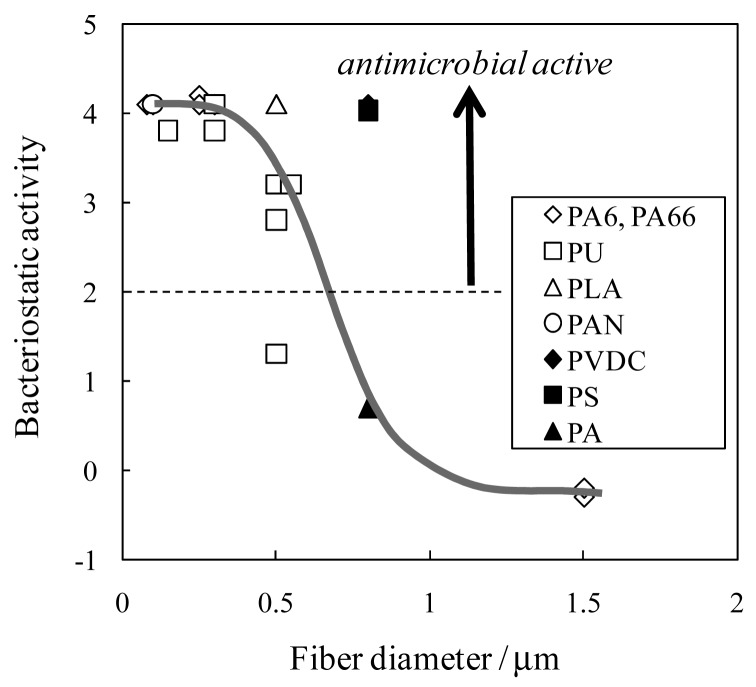
Relationship between fiber diameter and bacteriostatic activity (Adapted from [36]).

Interestingly, our recent experimental results revealed that many electrospun commodity polymer NFMs (e.g., poly(lactic acid) (PLA), polyamide (PA), polyurethane (PU), cellulose, polyacrylonitrile (PAN), polyvinylidene chloride (PVDC), and polystyrene (PS)) without the addition of an antimicrobial agent showed excellent antimicrobial properties (see Table 4). In particular, the antimicrobial properties of PAN, PVDC, and PS have not yet been reported. In addition, there is close correlation between the fiber diameter of NFMs and their antimicrobial activity (Figure 12); the NFMs with an average fiber diameter of less than 800 nm showed a better antimicrobial activity [36]. The mechanism of the antimicrobial properties has not been elucidated in detail. One possibility, however, may be the combination effect of the high surface area and the functional groups intrinsic to the respective polymers (e.g., carbonyl, amide, nitrile, halogen, and phenyl groups). These results indicate that electrospun polymer NFMs are a promising option as antimicrobial materials without using antimicrobial agents from commodity polymers.

**Table 4:** Antimicrobial activity of electrospun commodity polymer nanofibrous membranes (Adapted from [36]).

**Polymer**	**Chemical structure**	**Fiber diameter (nm)**	Bacteriostatic activity *1	Sterilization activity *2
Poly(lactic acid) (PLA)	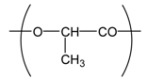	80,000 *3	0.2	−2.3
		500	4.1	1.5
Polyamide66 (PA66)	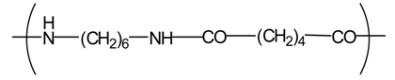	1,300	−0.2	−1.9
		250	4.2	1.6
Polyamide6 (PA6)	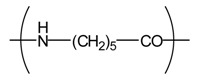	1,300	−0.3	−2.1
		300	4.1	1.5
Polyamide (PA)	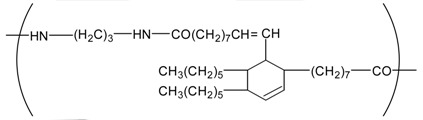	800	0.7	−2
Polyurethane (PU(ester))	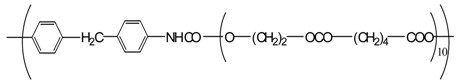	40,000 *3	−0.1	−1.9
		500	3.2	0.6
Polyurethane (PU(ether))	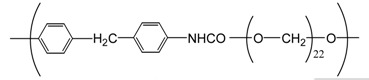	500	1.3	−1.4
Polyacrylonitrile (PAN)	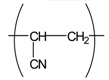	100	4.1	1.5
Polyvinylidene-chloride (PVDC)	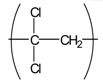	800	4.1	1.5
Polystyrene (PS)	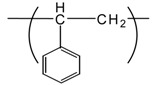	800	4.0	1.5

*1,2 Determined by Japanese Industrial Standard (JIS L1902) using *Staphylococcus aureus*

*1 Bacteriostatic activity = log N_2_ − log N_3_

*2 Sterization activity = log N_1_ - log N_3_; N_1_: initial cell number on control; N_2_: viable cell number on control after 18 h incubation. Cotton fabric is used as the control; N_3_: viable cell number on sample after 18 h incubation; Criteria of evaluation for bacteriostatic and sterilization activities are declared as follows: (1) If the bacteriostatic activity ≥ 2, the sample has antimicrobial activity; (2) If the sterilization activity ≥ 0, the sample has sterilization activity

*3 These thick fibers were prepared by wet spinning.

## 6. Summary and Future Directions

Electrospun nanofibrous membranes show unique functionalities based on their nanoscaled-size, high specific surface area, and highly molecular orientation. The combination of high surface areas of the nanofibers, surface chemistry and topology will provide improvement in the functions of the nanofibers (e.g., rapid kinetics of adsorption or ion exchange process, high adsorption or ion-exchange capacity, and high catalytic activity) and will lead to a significant expansion of nanofiber applications (e.g., high-speed water treatment, high capacity adsorbent, and highly effective catalyst). The functionalities of nanofibers and performances of nanofibrous membranes can be improved by controlling their fiber diameter, surface morphology, and internal structure of the nanofibers. These electrospun nanofibers or nanofibrous membranes are a promising material for the realization of a safe, sustainable, and healthy society.

Nanofiber technologies can lead to quantum leap innovations for the creation of materials with unique functions such as a nanosize effect, superhigh surface area, and molecular orientation effect. The mechanism of some unique functions of nanofibers (e.g., the fiber-diameter dependent antimicrobial effect and the high-adsorption ability of microparticles [37]) has not yet been fully elucidated. Since the research field of nanofibers contains both scientific and technological frontiers, it requires interdisciplinary backgrounds, such as physics, chemistry, polymer science, energy and environmental technologies, biological technology, electronics, and optics. In addition, we strongly believe that a large-scale production system with high-throughput and high-quality (*i.e.*, well-defined fiber morphology) tremendously facilitates the innovations in nanofibers.

## References

[1] Doshi J., Reneker D.H. (1995). Electrospinning process and applications of electrospun fibers. J. Electrost..

[2] Li D., Xia Y. (2004). Electrospinning of nanofibers: Reinventing the wheel?. Adv. Mater..

[3] Greiner A., Wendorff J.H. (2007). Electrospinning: A fascinating method for the preparation of ultrathin fibers. Angew. Chem. Int. Ed..

[4] Yoon K., Hsiao B.S., Chu B. (2008). Functional nanofibers for environmental applications. J. Mater. Chem..

[5] Matsumoto H. (2010). Nanofibrous membranes—Preparation and application of electrospun membranes. Maku.

[6] Son W.-K., Cho D., Park W.-H. (2006). Direct electrospinning of ultrafine titania fibers in the absence of polymer additives and formation of pure anatase titania fibers at low temperature. Nanotechnology.

[7] Zhou F.-L., Gong R.-H., Porat I. (2009). Mass production of nanofibre assemblies by electrostatic spinning. Polym. Int..

[8] Thompson C.J., Chase G.G., Yarin A.L., Reneker D.H. (2007). Effects of parameters on nanofiber diameter determined from electrospinning model. Polymer.

[9] Imaizumi S., Matsumoto H., Suzuki K., Minagawa M., Kimura M., Tanioka A. (2009). Phenolic resin-based carbon nanofibers prepared by electrospinning: Additive effects of poly(vinyl butyral) and electrolytes. Polym. J..

[10] Minato K., Ohkawa K., Yamamoto H. (2006). Chain conformations of poly(*γ*-benzyl-L-glutamate) pre and post an electrospinning process. Macromol. Biosci..

[11] Kakade M.V., Givens S., Gardner K., Lee K.H., Chase D.B., Rabolt J.F. (2007). Electric field induced orientation of polymer chains in macroscopically aligned electospun polymer nanofibers. J. Am. Chem. Soc..

[12] Kongkhlang T., Tashiro K., Kotaki M., Chirachanchai S. (2008). Electrospinning as a new technique to control the crystal morphology and molecular orientation of polyoxymethylene nanofibers. J. Am. Chem. Soc..

[13] Danno T., Matsumoto H., Nasir M., Shimizu S., Minagawa M., Kawaguchi J., Horibe H., Tanioka A. (2008). Fine structure of PVDF nanofiber fabricated by electrospray deposition. J. Polym. Sci. B Polym. Phys..

[14] Nakashima K., Tsuboi K., Matsumoto H., Ishige R., Tokita M., Watanabe J., Tanioka A. (2010). Control over internal structure of liquid crystal polymer nanofibers by electrospinning. Macromol. Rapid Commun..

[15] Imaizumi S., Matsumoto H., Konosu Y., Tsuboi K., Minagawa M., Tanioka A., Koziol K., Windle A. (2011). Top-down process based on electrospinning, twisting, and heating for producing one-dimensional carbon nanotube assembly. ACS Appl. Mater. Interfaces.

[16] Suzuki K., Matsumoto H., Minagawa M., Tanioka A., Hayashi Y., Fukuzono K., Gehan A.J., Amaratunga G.A.J. (2008). Carbon nanotubes on carbon fabrics for flexible field emitter arrays. Appl. Phys. Lett..

[17] Unalan H.E., Wei D., Suzuki K., Dalal S., Hiralal P., Matsumoto H., Imaizumi S., Minagawa M., Tanioka A., Flewitt A.J. (2008). Photoelectrochemical cell using dye sensitized zinc oxide nanowires grown on carbon fibers. Appl. Phys. Lett..

[18] Xie Y. (2008). Preparation and Characterization of Silica-Based Nanofibers by Electrospinning. M.Sc. Thesis.

[19] Streat M. (2004). Boom time for ion exchange. Chem. Ind. (London).

[20] Seo H., Matsumoto H., Hara S., Yako H., Minagawa M., Tanioka A., Yamagata Y., Inoue K. (2005). Preparation of polysaccharide nanofiber fabrics by electrospray deposition: Additive effects of poly(ethylene oxide). Polym. J..

[21] Matsumoto H., Wakamatsu Y., Minagawa M., Tanioka A. (2006). Preparation of ion-exchange fiber fabrics by electrospray deposition. J. Colloid Interface Sci..

[22] Imaizumi S., Matsumoto H., Ashizawa M., Tsuboi K., Minagawa M., Tanioka A. (2011). Preparation of ion-exchange carbon nanofibers by electrospinning: Effect of fiber diameter on their adsorption behaviors. Fiber Prepr. Japan.

[23] Matsumoto H., Yako H., Minagawa M., Tanioka A. (2007). Characterization of chitosan nanofiber fabric by electrospray deposition: Electrokinetic and adsorption behavior. J. Colloid Interface Sci..

[24] Matsumoto H., Nagata T., Minagawa M., Tanioka A. (2006). Preparation of polyelectrolyte nanofiber by electrospray deposition. Polym. Prepr. Japan.

[25] Yarin A.L., Zussman E., Wendorff J.H., Greiner A. (2007). Material encapsulation and transport in core–shell micro/nanofibers, polymer and carbon nanotubes and micro/nanochannels. J. Mater. Chem..

[26] Dong B., Gwee L., Salas-de la Cruz D., Winey K.I., Elabd Y.A. (2010). Super proton conductive high-purity Nafion nanofibers. Nano Lett..

[27] Kemperman A.J.B. (2000). Handbook on Bipolar Membrane Technology.

[28] Wakamatsu Y., Matsumoto H., Minagawa M., Tanioka A. (2006). Effect of ion-exchange nanofiber fabric on water splitting in bipolar membrane. J. Colloid Interface Sci..

[29] Brown R.C. (1993). Air Filtration: An Integrated Approach to the Theory and Applications of Fibrous Filters.

[30] Yamaguchi T., Shima T., Matsumoto H., Minagawa M., Tanioka A. (2010). Study on non-continuum air flow through electrospun nanofiber nonwovens. Fiber Prepr. Japan.

[31] Hinds W.C. (1998). Aerosol Technology.

[32] Pich J. (1971). Pressure characteristics of fibrous aerosol filters. J. Colloid Interface Sci..

[33] Nasir M., Matsumoto H., Minagawa M., Tanioka A., Danno T., Horibe H. (2007). Preparation of porous PVDF nanofiber from PVDF/PVP blend by electrospray deposition. Polym. J..

[34] Tan K., Obendorf S.K. (2007). Fabrication and evaluation of electrospun nanofibrous antimicrobial nylon 6 membranes. J. Membr. Sci..

[35] Matsumoto H., Minagawa M., Tanioka A., Yako H. Biological Ion-Exchange Nanofibre Fabrics by Electrospinning.

[36] Ogushi Y., Sasaki N., Imashiro Y., Minagawa M., Matsumoto H., Tanioka A. (2009). Antimicrobial activity of ultra-fine fiber nonwoven fabrics produced by electrospinning. Seikei Kakou.

[37] Graham K., Ouyang M., Raether T., Grafe T. Polymeric nanofibers in air filtration applications.

